# Meal Timing and Sleeping Energy Metabolism

**DOI:** 10.3390/nu15030763

**Published:** 2023-02-02

**Authors:** Rikako Yoshitake, Insung Park, Hitomi Ogata, Naomi Omi

**Affiliations:** 1 Graduate School of Comprehensive Human Sciences, University of Tsukuba, 1-1-1 Tennodai, Tsukuba 305-2628, Ibaraki, Japan; 2 International Institute for Integrative Sleep Medicine, University of Tsukuba, 1-2 Kasuga, Tsukuba 305-8550, Ibaraki, Japan; 3 Graduate School of Humanities and Social Sciences, Hiroshima University, 1-7-1 Kagamiyama, Higashi-Hiroshima 739-8521, Hiroshima, Japan; 4 Faculty of Health and Sport Sciences, University of Tsukuba, 1-1-1 Tennodai, Tsukuba 305-8574, Ibaraki, Japan

**Keywords:** meal timing, energy metabolism, sleep quality

## Abstract

There is a physiological link between sleep and eating. Insufficient sleep is a risk factor for overeating and excess body weight gain, and molecules such as orexin and insulin play a role in the control of sleep and energy intake. The effects of dietary timing on sleep and energy metabolism were examined in this review. First, we examined sleep energy metabolism and sleep quality under time-restricted eating, including skipping breakfast or dinner. Second, the mechanisms, benefits, and translational potential of the effects of time-restricted diets on sleep were discussed. Time-restricted eating under controlled conditions, in which daily caloric intake was kept constant, affected the time course of energy metabolism but did not affect total energy expenditure over 24 h. In free-living conditions, time-restricted eating for extended durations (4–16 weeks) decreased energy intake and body weight, and the effects of early time-restricted eating were greater than that of midday time-restricted eating. Although assessment of sleep by polysomnographic recording remains to be performed, no negative effects on the subjective quality of sleep have been observed.

## 1. Introduction

Eating and sleeping are two of the most time-consuming and life-supporting behaviors, which cannot be accomplished simultaneously. Sleep is linked to energy metabolism because regulatory factors such as orexin, leptin, and insulin affect both behaviors [1]. Differences in daytime eating patterns have been observed to affect the time course of energy metabolism during sleep. The cross-talk between the regulation of sleep and energy metabolism suggests a potential link between eating behavior and quality of sleep. A recent state-of-the-art study showed that sleep extension can reduce energy intake [2].

Most people have a monophasic sleep pattern (i.e., they become fully rested in one long sleep episode), and this extended period of fasting imposes a metabolic consequence. Modern society encourages convenient shortcuts provided by the industrial food economy, for example, “spinach in the freezer, canned wild salmon in the pantry, and commercial ravioli bought halfway around the world, to reduce cooking time. Somewhere between home-cooked food made from scratch or corporate fast food, we cook according to our location, day of the week, and mood” [3]. Moreover, we have a long period of time to eat throughout the day: from immediately after awakening to before bedtime. In developed societies, individuals largely eat throughout the wakeful hours [4]. Due to the effects of time-restricted eating on energy metabolism, it has been evaluated as an effective dietary intervention to prevent excess weight gain and related metabolic abnormalities. Skipping breakfast also restricts eating opportunities, but it is perceived as an unhealthy lifestyle. It is necessary to take into account the modality of time-restricted eating when evaluating its effect on health outcomes.

The present review discusses the effect of meal patterns on sleep. First, sleeping energy metabolism under time-restricted eating conditions, skipping breakfast, and skipping dinner is compared. Second, the relationship between sleeping energy metabolism and sleep quality is discussed. Finally, the effect of time-restricted eating on sleep, and its mechanisms, benefits, and translational potential are discussed.

## 2. Effect of Meal Pattern on Sleeping Energy Metabolism

People live by consuming energy through the intake of food and transforming it into energy that can be used by the body; daily energy consumption is divided into three categories: basal metabolic rate (60%), postprandial heat production (10–15%), and physical activity heat production (25–30%) [5]. In healthy people, the difference between energy intake and energy expenditure is kept low and constant, by balancing food intake and physical activity.

However, increased energy intake and decreased energy expenditure results in a relative excess of energy, causing excess energy to be stored in the body, mainly as triglycerides. In addition, energy expenditure during sleep is lower than during resting wakefulness, at about 90% of basal metabolism, and is not always constant during sleep but fluctuates with time [6]. Like fluctuations in blood glucose levels, energy expenditure is highest at the time of sleep onset, declines immediately after sleep onset, and then decreases with repeated slight increases and decreases, followed by a slight upward trend 1 to 2 h before awakening [6].

On the other hand, blood glucose levels increase with food intake, and the pancreas secretes insulin. This hormone lowers blood glucose levels, suppressing the increase in blood glucose levels. In addition, when muscle contractile activity associated with physical activity occurs, insulin causes more than 80% of blood glucose to bind to insulin receptors on skeletal muscle cell membranes, which is then taken up and used by skeletal muscle cells. Therefore, although blood glucose levels are homeostatically regulated, they change significantly with exercise and diet. In other words, blood glucose levels are an indicator of the type of physical activity occurring and dietary intake. Therefore, diet and physical activity strongly influence fluctuations in blood glucose and energy metabolism.

In a study [7] evaluating the effects of a single late evening meal on diurnal variation in blood glucose levels and energy expenditure over 24 h, the subjects stayed in a room-sized breathing chamber for 24 h. After entering the chamber at 17:00, the subjects had a normal (19:00) or late (22:30) dinner, breakfast and lunch, and remained in the chamber until 17:00 the next day. The changes in blood glucose levels were measured by a continuous glucose monitoring system. In another study [8] evaluating the effects of breakfast deprivation on energy metabolism and diurnal variation in blood glucose levels, the subjects entered the chamber at 22:00, went to bed at 23:00, and woke up at 7:00. They then had breakfast at 8:00 or skipped breakfast, had lunch at 12:00, and dinner at 19:00. The measurement period was 24 h from awakening (7:00), and the changes in blood glucose levels were measured by a continuous glucose monitoring system.

Of note, these studies all adopted an isocaloric meal protocol (i.e., the energy intake and macronutrient composition over 24 h were identical between the two dietary conditions) [7,8]. Late evening meals (19:00 vs. 22:30) [7] and large evening meals in the skipping breakfast condition (50% increase in caloric intake at dinner) [8] significantly increased blood glucose during sleep and over 24 h. Thus, changing meal times increases the average daily blood glucose level and the blood glucose level at night. In addition, changing meal times [7] or skipping breakfast [8] had no effect on total energy consumption over 24 h.

**Figure 1:**
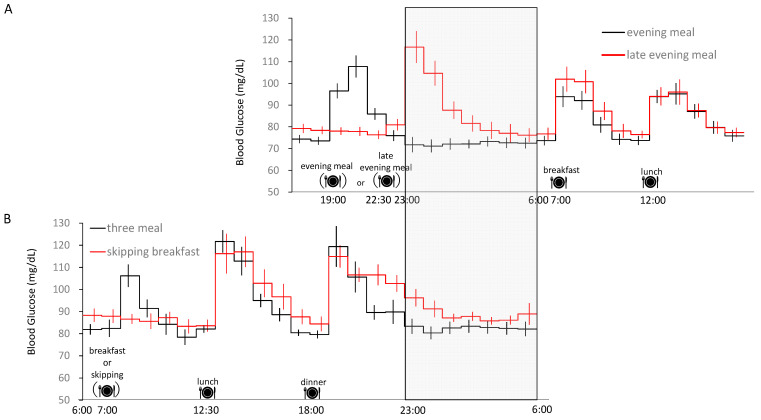
Twenty-four-hour blood glucose profile. Time course of blood glucose for (**A**) ordinary (19:00) and late evening meal (22:30) [7], and (**B**) skipping breakfast [9], as assessed by a continuous glucose monitoring system. Bedtime (23:00) and wake-up time (6:00) were identical in the two studies. The 24 h energy intake within each study was matched (i.e., the caloric intake of lunch and dinner was increased to compensate for the skipped breakfast). The gray area indicates sleep time, and bedtime (23:00) is shown in vertical rows. Partially modified from [7,9].

In another study [9] aiming to determine the effects of skipping breakfast for six consecutive days on energy metabolism and glycemic control, the subjects participated in two trials (three or two meals (skipping breakfast)) for six consecutive days. Blood glucose levels were measured using a continuous glucose monitoring system. When subjects skipped breakfast, they ate a large meal for lunch and dinner. Six days without breakfast significantly increased 24 h blood glucose levels, indicating that the period of time for which breakfast is skipped is also related to the regulation of 24 h blood glucose levels (Figure 1).

**Figure 2:**
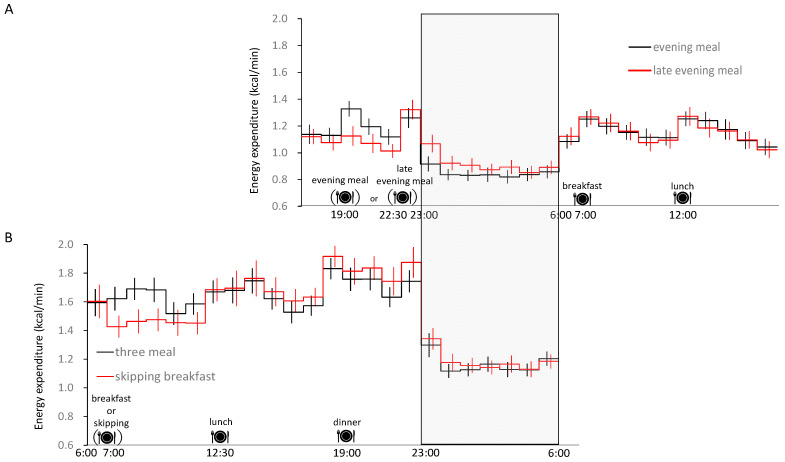
Twenty-four-hour blood glucose profile. Time course of energy expenditure for (**A**) ordinary (19:00) and late evening meal (22:30) [7], and (**B**) skipping breakfast [9], as assessed by whole-room indirect calorimetry. Bedtime (23:00) and wake-up time (6:00) were identical in the two studies. The 24 h energy intake within each study was matched (i.e., the caloric intake of lunch and dinner was increased to compensate for the skipped breakfast). The gray area indicates sleep time, and bedtime (23:00) is shown in vertical rows. Partially modified from [7,9].

In addition, skipping breakfast for six days had no effect on 24 h energy expenditure, similar to the results of studies with only one day of intervention [7,8]. Energy expenditure during sleep was significantly increased with a late evening meal [7]. On the other hand, energy expenditure was significantly increased when breakfast was skipped for only one day, but was not affected when breakfast was skipped for six days [9] (Figure 2). Respiratory quotient (RQ) generally increases with carbohydrate oxidation, but under skipping breakfast conditions, there was no effect on 24 h carbohydrate oxidation and RQ [8,9]. However, when breakfast was skipped for only one day, carbohydrate oxidation increased during sleep, and lipid oxidation decreased significantly [8], while six days of skipping breakfast had an effect on the time course of substrate oxidation during the day but not on carbohydrate or fat oxidation [9].

Skipping breakfast for six days altered the time course of diurnal substrate oxidation but had no significant effect on the accumulated oxidation of carbohydrate and fat over 24 h [9]. Even a single day of skipping breakfast decreased carbohydrate oxidation in the morning, followed by an increase in the evening and during sleep [8]. This finding does not support the hypothesis that skipping breakfast followed by large meals at lunch and dinner would reduce accumulated energy expenditure or fat oxidation over 24 h, and differed slightly from the findings of a recent German study, in which skipping breakfast caused an increase in 24 h energy expenditure [10]. The discrepancy between these studies is thought to be due to the gender of the subjects, the body mass index (BMI) of the subjects, the number of occasional breakfast skippers included in the studies, and the differing characteristics of the usual macronutrient balance of their habitual diets [9]. The German study evaluated blood glucose and energy expenditure under three conditions: skipping breakfast (BSD), skipping dinner (DSD), and the control (three meals). The subjects woke up at 6:00 and went to bed at 22:00. Meals were isocaloric (55% carbohydrate, 30% fat, and 15% protein), with breakfast (7:00), lunch (13:00), and dinner (19:00). When breakfast or dinner was skipped, subjects ate a large meal for lunch and dinner or breakfast. Blood glucose levels were measured by a continuous glucose monitoring system. As a result of the intervention, there was a blunted time course of energy expenditure, a lack of apparent thermic effect of food during the daytime, and a slower decline in sleeping energy expenditure compared with other studies, suggesting a delayed response in the indirect calorimetry used in their study. Fully understanding the effects of skipping breakfast on energy metabolism during sleep requires long-term intervention studies.

In young healthy subjects with dietary interventions such as large or late evening meals, there is greater carbohydrate oxidation during sleep [7,8,9], which results in higher blood glucose levels and continued metabolism of dietary sugars during sleep. In addition, diabetics have poor sleep quality [11], and treatment of sleep apnea in diabetics improves glycemic control [12]. In healthy individuals, sleeping for 4 h for one week increases insulin resistance and results in hyperglycemia similar to that seen in the early stages of diabetes [13]. Thus, sleep quality and glycemic control are closely related, and an increase in the size of evening meals due to skipping breakfast may decrease sleep quality.

The methods for measuring blood glucose include measurement by blood collection, blood glucose self-monitoring, and continuous blood glucose monitoring [14]. The continuous glucose monitoring system used in previous studies is a pager-sized device that records signals from a sensor inserted under the skin of the abdomen, and converts them into blood glucose readings every 5 min [15]. This is considered a useful tool for monitoring improvements in blood glucose control during ambulatory daily activities [16,17]. Therefore, this is also considered appropriate for measuring blood glucose changes over a long period of time in a restricted space, such as in a chamber. In recent years, some continuous glucose monitoring systems have also used enzyme electrodes to measure glucose concentrations in subcutaneous interstitial fluid, which do not require calibration [18,19]. However, continuous blood glucose monitoring systems must be chosen carefully, as some measuring devices can cause errors [20].

## 3. The Relationship between Energy Metabolism and Sleep

Many hormones and neuropeptides involved in the regulation of energy metabolism are also involved in the regulation of sleep/wakefulness. Ghrelin and orexin promote eating and arousal, whereas leptin and insulin promote satiety, enhance energy metabolism, and promote sleep [1]. An epidemiological study [21] examining the relationship between sleep duration and blood hormones found that blood leptin and ghrelin levels were lower and higher in short sleep durations, respectively. A study on two days of sleep restriction (4 h) in healthy subjects revealed a 28% increase in blood ghrelin levels, an 18% decrease in blood leptin levels, and a 23–24% increase in hunger and appetite, especially for high-calorie foods with high carbohydrate contents [22].

Carbohydrate intake activates a variety of brain areas involved in reward and motor activity regulation, and leads to short-term cognitive improvements [23], though it eventually leads to enhanced sleepiness and facilitates sleep onset [24,25,26,27].

The effects of high-glycemic index (GI) carbohydrate meals on sleep were evaluated using standard isocaloric meals with the same macronutrient composition. A significant reduction in the mean sleep onset latency was observed with the high-GI (9.0 ± 6.2 min) compared with the low-GI (17.5 ± 6.2 min) meal consumed 4 h before bedtime [26]. A possible mechanism behind the sleep-promoting effects of carbohydrate intake is direct stimulation by glucose of the ventrolateral preoptic nucleus in the hypothalamus, which plays a crucial role in the induction and maintenance of slow-wave sleep (SWS) [28]. Alternatively, the effects of elevated systemic glucose levels on sleep may be mediated by the effects of insulin on the central nervous system. When infused into the cerebral ventricular system, insulin promotes SWS, and intracerebroventricular infusions of polyclonal anti-insulin antibody results in decreased SWS [29]. Furthermore, elevated glucose and the subsequent insulin secretion increase the delivery of circulating tryptophan to the brain and its conversion to serotonin, which promotes sleep [30]. Large neutral amino acids (LNAAs, including tyrosine, phenylalanine, leucine, isoleucine, valine, and methionine) compete with tryptophan for blood–brain barrier transport [31]. Insulin promotes the selective uptake of LNAAs by muscle [26], reducing circulating levels of LNAAs and stimulating delivery of tryptophan to the brain. The mechanism behind the effect of high-GI carbohydrate meals on sleep remains to be elucidated.

## 4. Effects of Time-Restricted Eating on Sleep, and Its Mechanisms, Benefits and Translational Potential

When the daily eating window (>14 h) of overweight individuals was restricted to a self-selected 10 h for 12 weeks, the first caloric intake was delayed by 2.09 h and the last caloric intake was advanced by 2.08 h. Time-restricted eating extended the time between awakening and first caloric intake (1.81 vs. 3.64 h), and between last caloric intake and sleep onset (1.53 vs. 4.02 h) [18]. Self-selected time-restricted eating for 10 h, for 12 [18] or 16 weeks [4], decreased daily caloric intake and body weight, and improved cardiometabolic health without adverse effects on sleep. The protocols for time-restricted eating in other studies are presented in Table 1.

Time-restricted eating did not affect quality of sleep, as assessed by the Pittsburgh Sleep Quality Index (PSQI) [4,32,33,34,35,36]. Considering that the percentage of calories consumed after 20:00 is 19.9% on average [4], all time-restricted eating studies shifted the end of the eating window to earlier than that of the control condition. Reducing body weight by caloric restriction for 1–2 years [37] is related to improved sleep quality, as assessed by the PSQI. It is possible that the somnogenic effect of eating, as discussed in a previous section, was reduced by the time-restricted eating protocols, particularly between 6:00 and 15:00 [35]. Several methods are used to assess sleep quality, including validated questionnaires such as the PSQI, polysomnography, and wrist actigraphy [38]. However, low correlations between objective and subjective assessments of sleep quality have been reported [39,40]. In addition, skilled personnel are needed to install polysomnography electrodes, though it should be noted that polysomnographic recording of sleep at home has become possible [41]. Although beneficial effects of time-restricted eating on sleep were not reported, no adverse effects on sleep were found. Rigorous studies are needed to assess the objective quality of sleep using polysomnography.

In all the studies listed in Table 1, daily caloric intake and body weight were decreased. Objective assessment of energy intake in real-life settings is difficult; the assessment of dietary intake depends on the subject’s memory, requires skilled investigators, depends on the accuracy of food composition tables, and is particularly prone to errors due to under- and overreporting and inter-day variations [42]. Therefore, as well as knowing the types, characteristics, and extent of measurement errors in dietary surveys, additional methods of estimation using blood, urine, hair, and other ecological indicators also need to be considered. Moreover, when subjects become aware that their usual diet will be assessed, they often intentionally change their diet compared with their daily routine [43,44]. To obtain unbiased assessments of daily energy intake, a recent study adopted a new approach. The average energy intake in free-living conditions for two weeks was assessed based on changes in body energy content using dual X-ray absorptiometry, and energy expenditure was assessed by the doubly labeled water method [2]. The effects of time-restricted eating on energy intake are yet to be assessed using this new approach. As well as suppressing energy intake, time-restricted eating likely increases energy expenditure.

A recent study reported that time-restricted eating could affect negative energy balance without reducing energy intake. Using an indirect calorimetry chamber, time-restricted eating was found to significantly increase fecal energy loss (~22.7%) and urine energy loss (~14.5%) in healthy young subjects (age 24 ± 2.3 years; BMI 21.9 ± 1.7 kg/m^2^), without changing total energy expenditure [45]. Time-restricted eating interventions have been suggested as an alternative dietary strategy for obesity prevention and weight loss programs. In humans, fecal energy loss can amount to ~2–9% of ingested calories [46]. However, the mechanisms underpinning this energy loss and its physiological relevance remain poorly understood. A previous study has shown that there was a larger stool calorie loss during calorie restriction compared with overfeeding, and during oral vancomycin treatment compared with placebo [47]. These findings suggests that nutrient absorption is sensitive to changing environments, and supports a possible causal role for the gut microbiome in dietary energy balance. Fecal and urinary energy loss was estimated by direct calorimetry, also known as bomb calorimetry, which assesses the increase in temperature due to sample oxidation. Alternatively, in an animal experiment, time-restricted eating mitigates obesity through adipocyte thermogenesis; thus, the creatine–phosphocreatine futile cycle in adipocytes is an essential mechanism that drives the metabolic benefits experienced during time-restricted eating [48]. The effects of time-restricted eating on diet-induced thermogenesis and the creatine–phosphocreatine futile cycle remain to be studied in humans.

**Figure 3:**
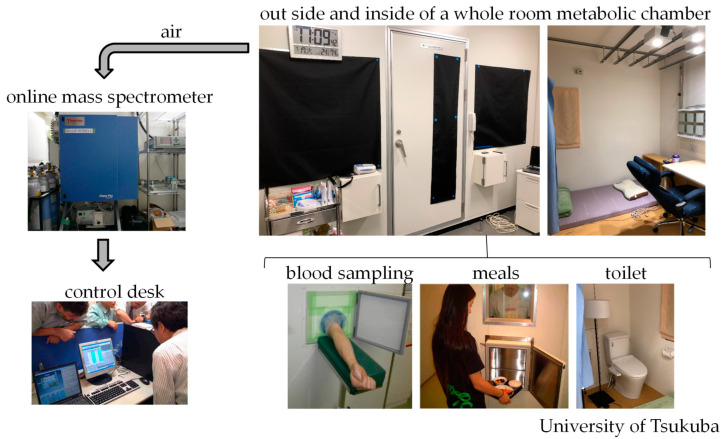
Whole-body indirect human calorimeter. A metabolic chamber for the measurement of substrate oxidation and energy expenditure over prolonged periods. In the airtight metabolic chamber (2.00 × 3.45 × 2.10 m; FHC-15S, Fuji Medical Science Co., Ltd., Chiba, Japan), air in the chamber was pumped out at a rate of 80 L/min. The temperature and relative humidity of the incoming fresh air was maintained at 25 °C and 55%, respectively. The chamber was furnished with an adjustable hospital bed, desk, chair, and toilet. Blood sampling and meal provision through the pass box did not affect the measurements. The concentrations of oxygen (O_2_) and carbon dioxide (CO_2_) in the outgoing air were measured with high precision by online process mass spectrometry (VG Prima PRO; Thermo Fisher Scientific, Winsford, UK). The precision of mass spectrometry, defined as the standard deviation of the continuous measurement of the calibrated gas mixture (O_2_, 15%; CO_2_, 5%), was <0.002% for O_2_ and CO_2_ [52]. O_2_ consumption ($\dot{V}$O_2_) and CO_2_ production ($\dot{V}$CO_2_) rates were calculated each minute, using an algorithm for improved transient response [53].

In the 19th century, direct calorimetry was developed to study human energy metabolism [49]. Currently, indirect calorimetry is almost exclusively used to measure energy metabolism and substrate oxidation in humans. Direct and indirect calorimetry are valuable methods for assessing energy consumption, but they do not measure the same energy. Indirect calorimetry estimates energy consumption based on measurements of oxygen consumption and carbon dioxide production. By contrast, direct calorimetry estimates energy consumption based on heat dissipation from the body [49]. For metabolic studies including meals and sleep, a whole-room metabolic chamber, which does not require the fitting of a mask or a mouthpiece to collect expired gas, is used [50]. In indirect calorimeters for whole-body use, the concentrations of gases in the air in the chamber are measured using online process mass spectrometry. These chambers are furnished with an adjustable bed, desk, chair, and toilet. Blood sampling and meal provision through a pass box do not affect the measurements (Figure 3). In our previous studies, energy expenditure and substrate oxidation [51] were calculated from O_2_ consumption ($\dot{V}$O_2_), CO_2_ production ($\dot{V}$CO_2_), and 24 h urinary nitrogen excretion (N, an index of protein oxidation that was assumed to be constant during calorimetry). The amounts of carbohydrates and fats oxidized are calculated using the following equations:

(1) $$\mathrm{Glucose}\;\mathrm{oxidation}(g/\min)=4.55\dot{V}{\mathrm{CO}}_{2}(L/\min)-3.21\dot{V}O_{2}(L/\min)-2.87N(g/\min)$$

(2) $$\mathrm{Fat}\;\mathrm{oxidation}(g/\min)=1.67\dot{V}O_{2}(L/\min)-1.67\dot{V}{\mathrm{CO}}_{2}(L/\min)-1.92N(g/\min)$$

Once the rates of glucose, lipid, and protein oxidation have been computed, the total rate of energy production can be estimated directly by taking into account the caloric equivalent of the three substrates [7]. The conversion factors for caloric equivalents are 4.10 kcal/g protein (25.625 kcal/g urinary nitrogen), 3.74 kcal/g carbohydrate, and 9.50 kcal/g fat [51].

The theoretical basis of indirect calorimetry was proposed by Ferrannini in 1988 [51]. It is necessary to clarify the difference between the gross energy of glucose and glycogen. The standard free energy change of glucose oxidation is 3.74 kcal/g. This value is smaller than that of glycogen (4.1 kcal/g) because there is relatively more carbon in 1 g of glycogen than in 1 g of glucose. The equation for the complete oxidation of glycogen is as follows:

(3) $$\mathrm{Glycogen}\;\mathrm{oxidation}(g/\min)=1.09\dot{V}{\mathrm{CO}}_{2}(L/\min)-2.88\dot{V}O_{2}(L/\min)-2.59N(g/\min)$$

Where there is reason to believe that tissue glycogen rather than glucose is the pre-dominant form of carbohydrate being oxidized, the equation of glycogen oxidation should be used. Having measured $\dot{V}$O_2_, $\dot{V}$CO_2_, and N, estimates of oxidized carbohydrate made based glucose oxidation or glycogen oxidation differ, although the difference is negligible in terms of energy expenditure. Thus, the estimation of energy expenditure is robust, even if the type of carbohydrate oxidized in the body is unknown. Finally, it is worth mentioning that the gross energy of glucose or glycogen for calculating energy expenditure should not be confused with the metabolizable energy of starch (4.0 kcal/g), which is referred to for calculating energy intake [7].

## 5. Implications and Future Directions

The advocates of time-restricted eating have mainly focused on early time-restricted eating, resulting in a long interval between the last meal of the day and bedtime [54]. In a study of 90 healthy subjects, early time-restricted eating (6:00–15:00) was more effective than midday time-restricted eating (11:00–20:00) in improving insulin sensitivity. Furthermore, early time-restricted eating improved fasting glucose, reduced total body mass and adiposity, ameliorated inflammation, and increased gut microbial diversity compared with midday time-restricted eating [35]. A survey of 53 pairs of twins who lived separately from their families revealed that the heritability of food timing varies by meal, and ranges from 56% for breakfast to 0% for dinner. The authors of the study suggested that intervening for the purpose of advancing late lunches and dinners may be more achievable than changing breakfast times [55]. The timing of dinner is likely to be influenced by individual lifestyles, but dinners consumed late at night have negative effects on metabolic health. For example, there is an increased risk of obesity [56,57], dyslipidemia [57], and diabetes [58], and insulin dysfunction and impaired glucose tolerance are induced [59]. The relationship between having late evening meals and missing breakfast also raises concerns about the impact of missing breakfast on quality of life, such as poor diet [60] and lack of physical activity [61].

Food components directly affecting sleep, such as tryptophan, are found in the diet, including in milk. After being metabolized, tryptophan becomes melatonin, followed by serotonin, which induces drowsiness [62]. Caffeine, found in coffee and tea, binds to adenosine receptors that promote drowsiness, thereby keeping us awake [63]. In adults, caffeine intakes above 100 mg have been reported to cause prolonged sleep latency and shortened sleep duration [64]. On the other hand, there are reports showing that caffeine intakes of less than 100 mg have no significant effect on sleep [65]. However, considering individual differences and sensitivities, even smaller amounts of caffeine may have ad-verse effects on sleep. Furthermore, caffeine intake affects energy expenditure, food intake, and sleep, as it causes thermogenesis [66]. There have been reports of decreased food intake [67,68] after caffeine intake. Many people consume coffee primarily in the morning and alcohol in the evening [4]. The underlying causes of the decrease in energy intake caused by early time-restricted eating in free-living conditions are related to changes in alcohol consumption. The effect of time-restricted eating on energy intake among subjects without a drinking habit, including children, remains to be clarified.

Of note, skipping breakfast and midday time-restricted eating each have distinct effects on the daily intake of food and nutrients. Controlled intervention trials under isocaloric conditions have shown little or no beneficial effect of breakfast on accumulated energy expenditure over 24 h [8,9,10,69,70,71]. In this regard, Kobayashi et al. [8] considered that decreasing meal frequency from 3 to 2 meals/day, or increasing it to 6, 7, or 14 meals/day [69,72,73,74], would have no acute effect on 24 h energy expenditure. In addition, Ohkawara et al. [70] found that increasing the number of meals from three to six per day had no significant effect on 24 h energy expenditure. However, increased meal frequency is likely to increase hunger and appetite, suggesting that increased meal frequency should be considered with an eye toward central nervous system function. Epidemiological studies have shown that skipping breakfast is associated with poor dietary quality [75], including lower total energy, vitamin, and mineral intake [76,77,78]. In addition, the time of onset of night-time melatonin secretion is accelerated by exposure to light during the day and by the intake of tryptophan, which is found in proteins sourced from breakfast meals [79]. Furthermore, insulin secretion resulting from eating breakfast has the effect of resetting the liver’s internal clock [80], suggesting that moderate carbohydrate and protein intake at breakfast can promote the resetting of the internal clock. Therefore, regular breakfast intake [81], one of the key indicators of a healthy lifestyle, should be recommended in order to have a fulfilling social life.

## Figures and Tables

**Table 1:** List of time-restricted eating studies.

Duration	Eating Window	Time	Sleep	Reference
16, 12 weeks	10 h	self-selected	PSQI didn’t change	[4,32]
8 weeks	4 h	15:00–19:00		[33]
8 weeks	6 h	13:00–19:00		[33]
12 weeks	8 h	10:00–18:00		[34]
5 weeks	9 h	6:00–15:00		[35]
5 weeks	9 h	11:00–20:00		[35]
4 weeks	8 h	12:00–20:00		[36] *

* Calorie-limited low carbohydrate. PSQI: Pittsburgh Sleep Quality Index.

## Data Availability

Not applicable.
